# Malformaciones esqueléticas y alteraciones del crecimiento en fetos de ratas con diabetes moderada

**DOI:** 10.7705/biomedica.5736

**Published:** 2021-09-22

**Authors:** Tahiry Gómez, Milagros García, Leticia Bequer, Cindy Freiré, María Aimee Vila, Sonia Clapés

**Affiliations:** 1 Unidad de Investigaciones Biomédicas, Universidad de Ciencias Médicas de Villa Clara, Santa Clara, Cuba Instituto Superior de Ciencias Médicas de Villa Clara Unidad de Investigaciones Biomédicas Universidad de Ciencias Médicas de Villa Clara Santa Clara Cuba; 2 Facultad de Medicina, Universidad de Ciencias Médicas de Villa Clara, Santa Clara, Cuba Instituto Superior de Ciencias Médicas de Villa Clara Facultad de Medicina Universidad de Ciencias Médicas de Villa Clara Santa Clara Cuba; 3 Instituto de Ciencias Básicas y Preclínicas "Victoria de Girón"' Universidad de Ciencias Médicas de La Habana, La Habana, Cuba Universidad de Ciencias Médicas de La Habana Instituto de Ciencias Básicas y Preclínicas "Victoria de Girón"' Universidad de Ciencias Médicas de La Habana La Habana Cuba

**Keywords:** diabetes mellitus experimental, teratogénesis, anomalías congénitas, macrosomía fetal, restricción del crecimiento fetal, Diabetes mellitus, experimental, teratogenesis, congenital abnormalities, fetal macrosomia, fetal growth retardation

## Abstract

**Introducción.:**

En la actualidad, la diabetes mellitus representa una de las condiciones médicas que complica el embarazo con mayor frecuencia, lo que afecta el crecimiento y el desarrollo fetal.

**Objetivo.:**

Determinar las malformaciones esqueléticas y alteraciones en el crecimiento en fetos de ratas Wistar diabéticas.

**Materiales y métodos.:**

Se utilizó un modelo de diabetes moderada inducida neonatalmente con estreptozotocina (STZ 100 mg/kg de peso corporal, por vía subcutánea) en ratas Wistar. En la adultez, las ratas sanas y diabéticas se aparearon con machos sanos de la misma edad y cepa. El día 20 de gestación se practicó la cesárea bajo anestesia. Se extrajeron los fetos, se pesaron y clasificaron como pequeños (PAG), adecuados (AEG) o grandes (GEG) para la edad gestacional. Los fetos seleccionados se procesaron para el análisis de anomalías esqueléticas y sitios de osificación.

**Resultados.:**

En la descendencia de las ratas diabéticas, hubo un mayor porcentaje de fetos clasificados como pequeños o grandes y un menor porcentaje de fetos con peso adecuado; el promedio de peso fetal fue menor y había menos sitios de osificación. Se observaron alteraciones en la osificación de cráneo, esternón, columna vertebral, costillas y extremidades anteriores y posteriores; y también, hubo una correlación directa entre el peso y el grado de osificación fetal.

Hubo malformaciones congénitas asociadas con la fusión y bifurcación de las costillas, así como cambios indicativos de hidrocefalia, como la forma de domo del cráneo, una amplia distancia entre los parietales y la anchura de las fontanelas anterior y posterior.

**Conclusión.:**

La diabetes moderada durante la gestación altera el crecimiento y el desarrollo fetal, que se ve afectado tanto por macrosomía y la restricción del crecimiento intrauterino como por malformaciones esqueléticas.

## Abstract (en)

**Introduction:**

Currently, diabetes mellitus represents one of the medical conditions that more frequently complicates pregnancy affecting the fetus's growth and development.

**Objective:**

To determine the skeletal malformations and growth alterations in fetuses of diabetic Wistar rats.

**Materials and methods:**

We used a neonatally streptozotocin-induced mild diabetes model (STZ 100 mg/kg body weight - subcutaneously) in Wistar rats. In adulthood, healthy and diabetic rats were mated with healthy males of the same age and strain. On day 20 of gestation, a cesarean was performed under anesthesia. Fetuses were removed, weighed, and classified as small (SPA), adequate (APA), and large (LPA) for the gestational age. Selected fetuses were processed for skeletal anomaly and ossification sites analysis.

**Results:**

In the offspring of diabetic rats, there was a higher percentage of fetuses classified as small or large and a lower percentage of fetuses with adequate weight; the fetal weight mean was lower and there were fewer sites of ossification.

Alterations were observed in the ossification of the skull, sternum, spine, ribs and fore and hind limbs; and also, there was a direct correlation between fetal weight and ossification degree There were congenital malformations associated with fusion and bifurcation of the ribs, as well as changes indicative of hydrocephaly, such as the dome shape of the skull, a wide distance between parietals, and the width of the anterior and posterior fontanels.

**Conclusion:**

Moderate diabetes during pregnancy alters fetal growth and development with macrosomia and intrauterine growth restriction, as well as skeletal malformations.

Según la Organización Mundial de la Salud (OMS), la diabetes se define como una enfermedad crónica grave que se desencadena cuando el páncreas no produce suficiente insulina o cuando el organismo no puede utilizar eficazmente la insulina que produce. La enfermedad se considera un problema de salud a nivel mundial debido a su gran incidencia y prevalencia ^1^. En 2019, se reportaron en el mundo 463 millones de personas adultas con diabetes mellitus y se estima que para el 2030 esta cifra aumentará a 578 millones ^2^.

La diabetes representa una de las condiciones médicas que con mayor frecuencia complica el embarazo. Las alteraciones en el control de la glucemia afectan a una de cada seis mujeres gestantes ^2^^,^^3^. En el 2019, más de 20 millones de nacidos vivos fueron hijos de madres con diabetes mellitus ^2^. Los fetos de madres diabéticas tienen características morfológicas, fisiológicas y patológicas diferentes debido a que se desarrollan en un ambiente intrauterino alterado ^4^. A pesar de los avances actuales en el cuidado de la mujer gestante con diabetes, la descendencia presenta hasta cinco veces mayor riesgo de alteraciones en el crecimiento ^5^^,^^6^ y malformaciones congénitas ^6^, siendo estas últimas una de las principales causas de muerte perinatal ^6^^,^^7^.

Las principales categorías de malformaciones observadas tanto en la diabetes clínica ^4^^,^^8^ como en la experimental ^9^ son las del sistema nervioso central, el cardiovascular y el musculoesquelético. Estas alteraciones se consideran secundarias a una diabetes mal controlada antes de la concepción o entre la tercera y la octava semanas de gestación, periodo embriogénico en que la mayoría de las mujeres gestantes desconocen su estado de gravidez ^6^^,^^10^.

La etiología, patogenia y prevención de las complicaciones inducidas por la diabetes requieren constantes esfuerzos de investigación básica y clínica ^11^. Los modelos animales de diabetes y gestación constituyen una importante herramienta para el estudio del desarrollo embrionario y fetal en la diabetes materna, dada la complejidad que implican estos estudios en humanos ^12^ o el empleo de métodos alternativos que no implican animales vivos ^9^.

Se han empleado diversos esquemas de inducción de la diabetes mellitus para intentar reproducir en animales los efectos de la diabetes moderada durante la gestación ^11^. Los resultados muestran que no siempre se logra obtener un patrón de daño como el que se desea evaluar en la descendencia. Esto se debe a las dificultades propias de un modelo experimental, en particular, cuando se pretende evaluar el crecimiento y el desarrollo de la primera generación ^11^^,^^13^. Con este estudio, nos propusimos estudiar las malformaciones esqueléticas y alteraciones en el crecimiento en fetos de 20 días descendientes de ratas Wistar con diabetes moderada.

## Materiales y métodos

### Diseño experimental

Se utilizaron ratas Wistar adultas vírgenes (180 ± 20 g de peso) de ambos sexos producidas en el Centro de Producción de Animales de Laboratorio (CENPALAB), en La Habana, Cuba. Para la obtención de las crías, se aparearon hembras y machos. Antes del apareamiento, las hembras se dividieron en dos grupos: aquellas a cuyas crías se les induciría la diabetes en el período neonatal y las del grupo de control. Al nacer, las camadas de las madres en cada grupo se colocaron en áreas separadas y debidamente identificadas. Se mantuvo en cada caja a las madres con las crías hembras de la camada, en tanto que los machos se cedieron para otros estudios en la universidad.

Se indujo diabetes en la crías hembras seleccionadas en el segundo día de nacidas (grupo D) mediante inyección subcutánea de estreptozotocina (STZ, Applichem, Alemania) en dosis de 100 mg/kg de peso corporal disuelta en solución tampón de citrato de sodio de 0,1 M y pH 4,5. El modelo aplicado permite la obtención de hiperglucemias moderadas en la adultez ^14^^,^^15^. Las crías del grupo control (grupo C) recibieron solución tampón de citrato sódico en similares condiciones. A todas las crías se les permitió crecer hasta los 120 días del nacimiento con suministro de agua y comida *ad libitum,* y condiciones controladas de temperatura de 19 a 25 °C, humedad relativa de 45 a 65 % y ciclos de 12 horas de luz y oscuridad, como se establece para este tipo de experimento. El protocolo fue aprobado por el Comité de Ética de la Investigación de la Universidad de Ciencias Médicas de Villa Clara y se ajustó a la "Guía para el cuidado y uso de animales de laboratorio" ^16^.

Ambos grupos de ratas (D y C) se aparearon con machos sanos de la misma edad y cepa. La mañana en que se observaron espermatozoides en el lavado vaginal se consideró el día cero de preñez; la glucemia se determinó mediante el uso de un glucómetro y biosensores SUMA a partir de una gota de sangre de la punta de la cola. Se incluyeron diez ratas preñadas en el grupo de control y diez en el grupo experimental con diabetes, y se colocaron en cajas independientes en el área de gestación bajo estricta vigilancia y seguimiento metabólico hasta el día en que se practicó la eutanasia.

### Cesárea y eutanasia, selección de los fetos y análisis morfológico

El día 20 de gestación se practicó la cesárea bajo anestesia con 50 a 60 mg/kg de tiopental sódico (Farmahealth-Laboratories Rotifarma-SA, India). La eutanasia se practicó por desangrado mediante punción intracardiaca. Se extrajeron ambos cuernos uterinos y, después de extirpar la grasa y el tejido periuterino, se obtuvieron los fetos.

El peso de todos los 193 fetos vivos se determinó en una balanza digital con una sensibilidad de 0,01 g (YAMATO, China): 105 descendientes de ratas sanas y 88 de ratas diabéticas. Los fetos fallecidos se excluyeron de la investigación y, del total de fetos vivos, se seleccionó un tercio de cada camada para el examen esquelético; en total, 58 fetos: 28 descendientes de ratas sanas y 30 de ratas diabéticas.

Los fetos se clasificaron según los valores percentiles del peso fetal del grupo control, siguiendo la metodología de Soulimane-Moktari, *et al.*^17^, de la siguiente manera: pequeño para la edad gestacional (PEG), feto con peso menor que el valor del percentil 5; adecuado para la edad gestacional (AEG), feto con peso entre los valores de los percentiles 5 y 95, y grande para la edad gestacional (GEG), feto con peso mayor que el valor del percentil 95.

El procedimiento para el examen esquelético consistió en la transparentación de especímenes y tinción ósea, descrito por Staples, *et al.*^18^. La observación se realizó con estereoscopio (BMS, EE. UU.) y se documentó del con una cámara fotográfica (Canon, Japón). Se analizaron los huesos del cráneo, y las vértebras cervicales, torácicas, lumbares, sacras y caudales, las costillas, el esternón (manubrio, xifoides y centros esternales), los miembros anteriores (carpos, metacarpos y falanges), los miembros posteriores (tarsos, metatarsos y falanges) y la pelvis. Se tuvieron en cuenta el tamaño, la forma, el número, la posición, la adición, la ausencia o la fusión de huesos, así como el grado de osificación ^19^. Todo el procedimiento se llevó a cabo según los parámetros propuestos por Aliverti, *et al.*^20^.

### Análisis estadístico

Debido a la distribución no gaussiana de las variables cuantitativas, se utilizó la prueba no paramétrica U de Mann Whitney para determinar si existían diferencias entre los grupos. La prueba exacta de Fisher se empleó para comparar los valores porcentuales y la relación entre las variables se determinó mediante la correlación de Spearman. Las diferencias se consideraron estadísticamente significativas cuando p era menor de 0,05.

## Resultados

En el día inicial de la gestación, la glucemia presentó valores significativamente superiores (p<0,05; Mann-Whitney) en las ratas diabéticas (7,65 ± 0,16 mM) comparadas con las sanas (5,14 ± 0,09 mM).

Los fetos de ratas diabéticas presentaron un menor peso promedio a los 20 días de gestación en comparación con los descendientes de ratas sanas. El grupo diabético registró un mayor porcentaje de fetos PEG y GEG, y uno menor de AEG con respecto al grupo de control. En la descendencia de las ratas sanas, predominaron los fetos AEG (cuadro 1).

**Cuadro 1:** Variables de crecimiento y desarrollo en fetos de ratas diabéticas (D) y controles (C)

Variables	Grupos	
	C	D
Peso y clasificación acorde al peso fetal		
Fetos examinados^a^	105	88
Peso^b^	3,70 ± 0,050	3,56 ± 0,071^*^
PEG^c^	5 (4,8)	14 (15,9)^&^
AEG^c^	97 (92,3)	65 (73,9)^&^
GEG^c^	3 (2,9)	9 (10,2)^&^
Alteraciones esqueléticas		
Fetos examinados^a^	28	30
Cráneo^c^		
Osificación parcial del frontal	1 (3,57)	3 (10,00)
Osificación parcial de los parietales	4 (14,29)	22 (73,33)^&^
Osificación parcial del interparietal	0 (0,00)	12 (40,00)^&^
Osificación parcial del supraoccipital	0 (0,00)	2 (6,67)
Esternón^c^		
Osificación parcial de la primera esternebra	0 (0,00)	3 (10,00)
Osificación parcial de la tercera esternebra	0 (0,00)	1 (3,33)
Osificación parcial de la cuarta esternebra	2 (714)	11 (36,67)^&^
Ausencia de la cuarta esternebra	0 (0,00)	3 (10,00)
Osificación parcial del xifoides	0 (0,00)	1 (3,33)
Ausencia del xifoides	1 (3,57)	5 (16,67)
Región caudal^c^		
Osificación parcial de primera vértebra caudal	0 (0,00)	1 (3,33)
Osificación parcial de segunda vértebra caudal	0 (0,00)	1 (3,33)
Osificación parcial de tercera vértebra caudal	0 (0,00)	1 (3,33)
Ausencia de la cuarta vértebra caudal	0 (0,00)	3 (10,00)
Ausencia de la quinta vértebra caudal	8 (28,57)	14 (46,67)
Ausencia de la sexta vértebra caudal	16 (57,14)	30 (100,00)^&^
Patas anteriores^c^		
Ausencia del cuarto metacarpo	3 (10,71)	10 (33,33)^&^
Ausencia de falanges	2 (714)	8 (26,67)
Patas posteriores^c^		
Ausencia del quinto metatarso	0 (0,00)	1 (3,33)
Ausencia de falanges	1 (3,57)	9 (30,00)^&^
Sitios de osificación		
Fetos examinados^a^	28	30
Esternón"	5,89 ± 0,08	5,20 ± 0,19^*^
Vertebras caudales^b^	5,14 ± 0,16	4,33 ± 0,19^*^
Metacarpos^b^	3,89 ± 0,06	3,67 ± 0,09^*^
Metatarsos^b^	5,00 ± 0,00	4,97 ± 0,03
Falanges anteriores^b^	3,71 ± 0,20	2,93 ± 0,33^*^
Falanges posteriores^b^	3,86 ± 0,14	2,80 ± 0,34^*^
Total de sitios de osificación^b^	27,49 ± 0,42	23,90 ± 0,90^*^

PEG: pequeño para la edad gestacional, AEG: adecuado para la edad gestacional, GEG: grande para la edad gestacional

^a^ Datos presentados como total

^b^ Datos presentados como media ± error estándar de la media. * Diferencias significativas con respecto al grupo de control (p<0,05, prueba U de Mann-Whitney)

^c^ Datos presentados como total y porcentaje. & Diferencias significativas con respecto al grupo control (p<0,05, prueba exacta de Fischer)

Los fetos descendientes de ratas diabéticas mostraron menor grado de osificación en el cráneo, el esternón, la columna vertebral y las extremidades anteriores y posteriores, que la descendencia de las ratas de control (cuadro 1). El resto de las regiones del esqueleto se encontraron completamente osificadas en los descendientes de ambos grupos.

**Figura 1:**
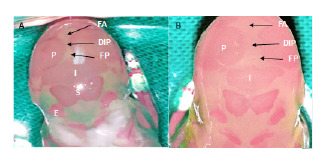
Alteraciones en la osificación de los huesos del cráneo. **A:** Feto del grupo control. Osificación completa de los huesos parietales (P), interparietales (I), supraoccipitales (S) y exoccipitales (E). Dimensiones normales de la fontanela anterior (FA), la fontanela posterior (FP) y la distancia interparietal (DIP). **B:** Feto del grupo diabético. Osificación parcial de los huesos parietales e interparietales. Fontanelas anterior y posterior anchas, distancia interparietal amplia, cráneo en forma de domo

En la descendencia de madres diabéticas, se encontró retardo en la osificación en cuatro de los siete huesos pertenecientes a la bóveda craneana. En los huesos parietales, interparietales, frontal y supraoccipital, se detectó un mayor retardo en la osificación en los fetos del grupo diabético que en los de control, aunque en los dos últimos huesos el retardo en la osificación no fue significativo (cuadro 1 y figura 1). Además, en seis descendientes de madres diabéticas, que representaban el 20 % del total del grupo, se identificaron alteraciones morfológicas asociadas con la osificación parcial de los huesos parietales, alteraciones descritas como amplia distancia entre estos huesos y fontanelas anterior y posterior anchas, lo que le confirió forma de domo al cráneo de estos fetos (figura 1).

**Figura 2:**
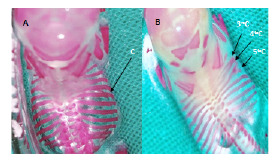
Malformaciones en costillas. **A.** Feto del grupo control. Osificación completa de las costillas (C). **B.** Feto del grupo diabético. Tercera y cuarta costillas fusionadas, quinta costilla bifurcada

En la región de las costillas, se detectaron malformaciones en un descendiente de rata diabética con presencia de bifurcación en la quinta costilla derecha y fusión en el extremo distal de las tercera y cuarta costillas derechas (figura 2).

**Figura 3:**
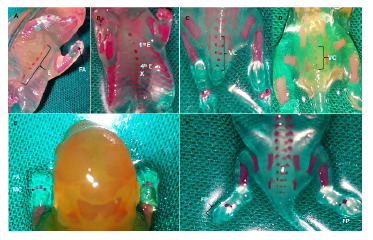
Alteraciones en la osificación de esternón, columna vertebral y extremidades. Sitios de osiicación. **A:** Esternón en feto del grupo control. Osiicación completa de los seis centros del esternón (E) y falanges anteriores (FA). **B:** Esternón en feto del grupo diabético. Osiicación parcial de la primera esternebra (1^ra^ E), ausencia de la cuarta esternebra (4^ta^ E) y ausencia del xifoides (X). **C:** Vértebras caudales en feto del grupo control. Osiicación completa de las vértebras caudales (VC). **D:** Vértebras caudales en feto del grupo diabético. Ausencia de osiicación de arcos en las vértebras caudales con osiicación completa de tres centros de las vértebras caudales. **E:** Huesos de las patas anteriores en feto del grupo diabético. Ausencia de falanges anteriores (FA) y cuarto metacarpo (MC). **F:** Huesos de las patas posteriores en feto del grupo diabético. Ausencia de falanges posteriores (FP)

En la región del esternón, se evidenció retardo en la osificación de la primera, tercera y cuarta esternebras en los fetos del grupo diabético. La ausencia de la cuarta esternebra no fue significativa, sin embargo, la osificación parcial o la ausencia de este hueso se observó en el 46,67 % de los descendientes de ratas diabéticas. La ausencia del xifoides se presentó en cinco fetos del grupo de ratas diabéticas, en tanto que se observó solo en un feto del grupo de control. La osificación parcial del xifoides se evidenció en un descendiente de madre diabética, lo que representa un 20 % de los fetos de dicho grupo (cuadro 1 y figura 3 A y B). El número de vértebras caudales fue variable; no obstante, en el grupo de ratas diabéticas se observó un número mayor de fetos con ausencia de las vértebras quinta y sexta (cuadro 1 y figura 3 C y D). En los descendientes de madres diabéticas, se evidenció retardo en la osificación de los huesos de las patas anteriores y posteriores. En las patas anteriores, el cuarto metacarpo y las falanges, el retardo en la osificación era significativo, así como la ausencia de falanges en las patas posteriores (cuadro 1 y figura 3 E y F).

En cuanto a los sitios de osificación, se encontraron carencias en el esternón, las vértebras caudales, los metacarpos y las falanges anteriores y las posteriores de los fetos de ratas diabéticas, con respecto a los descendientes de las ratas de control. Un comportamiento similar se presentó en todos los sitios de osificación (cuadro 1 y figura 3).

**Figura 4:**
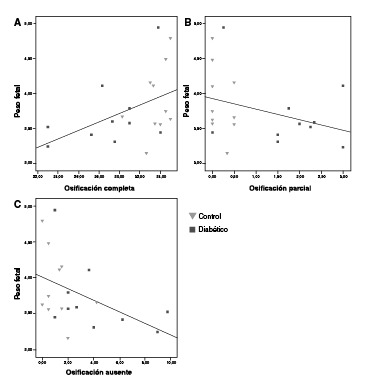
Correlación entre el grado de osificación y el peso fetal. A: Osificación completa *Vs.* peso fetal (p=0,469; p=0,037*). B: Osificación parcial *Vs.* peso fetal (p=-0,333; p=0,151). C: Osificación ausente *Vs.* peso fetal (p=-0,518; p=0,019*) (prueba de rango de correlaciones de Spearman)

El peso fetal se relacionó con el grado de osificación, pues los fetos con mayor número de huesos completamente osificados alcanzaron mayor peso fetal y viceversa (figura 4 A, B, C).

## Discusión

En varios estudios en modelos de roedores de diabetes moderada y gestación, se han evidenciado condiciones adversas de mediana magnitud que conducen a consecuencias de diversa índole sobre la descendencia ^9^^,^^11^.

En este experimento, los niveles de glucosa en ayuno fueron más altos en el grupo de ratas diabéticas que en las de control, con valores discretamente superiores a 7 mM, lo que permitió verificar la presencia de hiperglucemias moderadas en el día inicial de la gestación. La mayor frecuencia de fetos sin el peso adecuado para la edad gestacional en el grupo de ratas diabéticas, indicó la incidencia de restricción del crecimiento intrauterino, así como de macrosomía, resultados análogos a los reportados en modelos de diabetes moderada y gestación ^13^^,^^21^^,^^22^.

La regulación del crecimiento fetal en la gestación diabética es un proceso complejo que depende de varios factores, como el tipo de diabetes, la disponibilidad de nutrientes y oxígeno para el feto, una variedad de factores de crecimiento de origen materno, fetal y placentario, y la unidad funcional madre-placenta-feto ^4^^,^^23^^,^^24^. Los daños generados por el entorno intrauterino hiperglucémico en la placenta afectan su estructura morfológica y su función ^11^ y pueden inducir desviaciones en el peso que afectan de manera diferente a los fetos de una misma camada ^9^. Las alteraciones en la placenta pueden provocar restricción del crecimiento fetal por condiciones hipóxicas y afectaciones en el transporte de nutrientes ^5^, en tanto que pueden generar macrosomía cuando existe mayor transferencia de nutrientes asociada con elevados niveles de metabolitos circulantes en la madre y al límite que posee la placenta para acumular los excesos de sustratos de origen materno ^17^^,^^23^. Los datos aportados en varios estudios de placenta en diabetes materna, sugieren una elevada incidencia de inmadurez placentaria comparada con la de embarazos normoglucémicos ^25^. Aunque en el presente estudio no se analizó el estado de la placenta, es importante destacar que los fetos de ratas diabéticas presentaron menor eficiencia placentaria (calculada como peso fetal dividido por peso placentario) que los descendientes de ratas sanas.

Los patrones de osificación son similares en el humano y en la rata, los huesos se originan por osificación membranosa o endocondral, procesos controlados por factores genéticos y ambientales ^26^. El esqueleto fetal se considera un indicador útil del desarrollo embrionario al reflejar las alteraciones del ambiente intrauterino ^26^^,^^27^. En fetos de ratas con enfermedades de origen metabólico, como la diabetes, se ha comprobado el retardo en la osificación del esqueleto ^21^^,^^22^.

En el presente estudio, se comprobó el retardo en el proceso de osificación en varios de los huesos analizados. Los fetos de ratas diabéticas presentaron alteraciones en los huesos del cráneo, con retardo en la osificación de los parietales, los interparietales, el frontal y el supraoxipital. Estos resultados, analizados en conjunto con los hallazgos de mayor distancia entre los parietales y mayor amplitud de las fontanelas anterior y posterior, así como la forma de domo en el cráneo, podrían constituir una novedad del presente estudio. Los trabajos revisados en modelos de diabetes moderada no reportan modificaciones en la osificación en los huesos de la bóveda craneana de la descendencia ^21^^,^^22^^,^^28^.

Las alteraciones en la osificación del esternón antes mencionadas corresponde con lo reportado por otros autores en modelos experimentales de diabetes moderada ^21^^,^^22^. Se ha observado que, en el día 20 de gestación, varias esternebras y el xifoides aún no se han osificado y que, en el día 21, su osificación es completa. Este trastorno parece ser transitorio, pues hay estudios que corroboran que se produce una recuperación posterior durante el período neonatal ^20^^,^^29^.

La correlación directa entre el grado de osificación y el peso de los fetos estuvo en concordancia con reportes previos ^20^^,^^26^, lo que indica que el peso fetal se ve afectado cuando no se ha completado el proceso de osificación. El retraso en la osificación en diferentes regiones del esqueleto, acompañado del menor número de sitios de osificación, contribuyó a un mayor porcentaje de fetos PEG en el grupo de ratas diabéticas, confirmando que la gestación diabética puede causar en los fetos retardo en el crecimiento además de macrosomía. Resultados similares se han reportado en este modelo experimental en un estudio en el que las ratas se gestaron a los 90 días de evolución de la diabetes ^13^, así como en otros modelos de diabetes moderada ^21^^,^^22^^,^^28^.

Entre los mecanismos involucrados en el efecto teratogénico de la hiperglucemia, se encuentran el estrés oxidativo y el estrés nitrosativo. Ambos pueden modificar múltiples vías de señalización, y provocar daño celular masivo y eventos apoptóticos que conduce a defectos en el desarrollo embrionario y fetal ^10^^,^^30^. El estudio del desarrollo de las estructuras cráneo-faciales ha aportado pruebas de que la diabetes durante la gestación ejerce un efecto teratogénico sobre las células de la cresta neural cefálica, precursoras de los huesos de la cara y la bóveda craneana, afectando su migración y proliferación ^26^^,^^31^^,^^32^. Se ha propuesto que la hiperglucemia provoca un desequilibrio oxidativo que conlleva a un silenciamiento en la expresión del gen *Pax3,* por lo que se produce un incremento de la apoptosis. Esta situación induce modificaciones en la migración de las células de la cresta neural, de lo que se derivan defectos en el cierre del tubo neural y en otras estructuras del cerebro, entre otras anomalías ^6^^,^^31^^,^^32^.

En los fetos descendientes de ratas diabéticas del presente estudio, el conjunto de características morfológicas apreciadas en la región cráneo-facial es atribuible a posibles daños sobre las células de la cresta neural provocados por la diabetes materna. Estos hallazgos constituyen signos de dilatación moderada o extrema de los ventrículos laterales en el cerebro que podrían sugerir la presencia de hidrocefalia, una de las malformaciones del sistema nervioso central regularmente observada en la diabetes materna clínica y experimental ^32^.

Vale la pena señalar que los fetos portadores de alteraciones en las regiones del cráneo y las costillas fueron descendientes de ratas diabéticas con descontrol glucémico antes y durante la gestación. El hallazgo de las malformaciones fetales en este estudio coincide con investigaciones previas de este equipo de trabajo en las que las malformaciones externas fetales se asociaron con el descontrol metabólico materno y la disminución de la actividad antioxidante en tejidos fetales y placentarios ^13^. El incremento de la incidencia de malformaciones esqueléticas en fetos de ratas diabéticas podría asociarse al efecto teratogénico del ambiente intrauterino hiperglucémico, lo que permite comprobar que varias alteraciones provocadas por la diabetes en la madre, aun con hiperglucemias moderadas, producen resultados adversos antes y después del nacimiento.

Se concluyó que la diabetes moderada durante la gestación alteró el crecimiento y el desarrollo fetal, caracterizados tanto por macrosomía y restricción del crecimiento intrauterino, como por malformaciones esqueléticas. Aunque las principales limitaciones de este estudio se relacionan con las diferencias entre la gestación diabética experimental y la humana, los resultados son comparables a lo observado en descendientes de mujeres con diabetes materna, en los que el desarrollo prenatal se ve afectado y el índice de malformaciones se incrementa ^11^^,^^30^ Este modelo experimental da oportunidades para estudiar intervenciones farmacológicas, que tempranamente aplicadas, permitirían mejorar la salud de futuras generaciones.
